# Ocular biometric changes following unilateral cataract surgery in children

**DOI:** 10.1371/journal.pone.0272369

**Published:** 2022-08-05

**Authors:** Yooyeon Park, Hae Ri Yum, Sun Young Shin, Shin Hae Park

**Affiliations:** 1 Department of Ophthalmology, College of Medicine, Dankook University, Cheonan, Republic of Korea; 2 Department of Ophthalmology, Eunpyeong St. Mary’s Hospital, College of Medicine, The Catholic University of Korea, Seoul, Republic of Korea; 3 Department of Ophthalmology, Seoul St. Mary’s Hospital, College of Medicine, The Catholic University of Korea, Seoul, Republic of Korea; University of Warmia, POLAND

## Abstract

**Purpose:**

To analyze ocular biometric changes following unilateral cataract surgery in children.

**Methods:**

A total of 57 children aged under 13 years who underwent unilateral cataract surgery were analyzed. Groups were classified according to their age at surgery: group I (age <3), II (3≤ age <6), III (6≤ age <9), and IV (age ≥9). The myopic shift, axial growth, and corneal curvature changes were compared between the pseudophakic eyes and the fellow phakic eyes.

**Results:**

During 7.81 ± 4.39 years, the overall myopic shift (D) and the rate of myopic shift (D/year) were significantly higher at -3.25 ± 3.21 D and -0.45 ± 0.44 D/year in the pseudophakic eyes than -1.78 ± 2.10 D and -0.22 ± 0.29 D/year in the fellow phakic eyes (P = 0.01, 0.004). Group I (-1.14 ± 0.66 vs -0.02 ± 0.45 D/year) and group II (-0.63 ± 0.37 vs -0.31 ± 0.29 D/year) showed significantly higher rate of myopic shift in the pseudophakic eyes than in the phakic eyes. The rate of myopic shift in the pseudophakic eyes decreased in the older age groups (P = 0.001). There was no significant between-eye difference in the changes in axial length and keratometric values postoperatively.

**Conclusion:**

Following unilateral cataract surgery, a significant postoperative myopic shift was noticed in the pseudophakic eyes compared to the fellow phakic eyes in groups under 6 years old. Postoperative myopic shift and the resultant anisometropia should be considered when selecting the optimal power of IOL in young children requiring unilateral cataract surgery.

## Introduction

Cataracts are one of the major preventable causes of childhood amblyopia, which is preventable by early intervention before severe visual deprivation amblyopia occurs [1]. The implantation of an intraocular lens (IOL) in children undergoing cataract surgery has become widely accepted and performed at a younger age with the development of surgical instruments and techniques [2–17]. Despite the increasing frequency of IOL implantation in children, IOL selection is still challenging due to normal ocular growth, poor cooperation with biometric measurements, and the lack of a biometric formula for small-sized pediatric eyes. In contrast to adult cataract surgery, it is important to understand that children do not have a static course after cataract extraction. As pediatric eyes grow normally, the axial length (AL) typically grows, the cornea flattens, the anterior chamber gets deeper, lens thickness decreases, and vitreous chamber depth increases with age, which keeps the refractive status constant [18–20]. Rapid axial elongation occurs during the first 2 years of life and slows and stabilizes until the end of the first decade [19]. However, since cataract surgery consists of removing the lens, changes in corneal curvature alone cannot offset the effect of ocular growth. It is thought to undergo a rapid myopic shift after surgery [21–25]. Based on the anticipated development of a myopic shift after cataract surgery, it has been generally accepted to target the postoperative refraction to an initial hyperopia rather than to emmetropia, although the amount of undercorrection varies depending upon age [26]. Moreover, a myopic shift could be a more difficult issue to deal with in children with unilateral pseudophakia, as significant postoperative anisometropia following unilateral cataract surgery can lead to amblyopia and impaired binocularity.

The analysis of a postoperative myopic shift developing after pediatric cataract surgery could help set the target refraction at the time of IOL implantation more accurately and provide guidelines for the long-term care for vision development. Unfortunately, there have been a few studies regarding long-term refractive changes over 2 years in children with unilateral pseudophakia. In addition, the results from studies with Western populations should be applied with caution in Korean children, in which a higher prevalence and severity of myopia have been reported [27]. We sought to analyze ocular biometric changes and postoperative myopic shifts following unilateral cataract surgery in children of different age groups.

## Materials and methods

This retrospective study was performed according to the principles of the Declaration of Helsinki after approval was granted by the Institutional Review Board (IRB) of Seoul St. Mary’s Hospital, College of Medicine, the Catholic University of Korea, Seoul, Korea (KC21RISI0136). The IRB waived the requirement for informed consent and all data were fully anonymized before accessed.

The medical records of 90 children under the age of 13 years who were clinically confirmed to have cataracts and underwent cataract surgery with IOL implantation in a unilateral eye at Seoul St. Mary’s Hospital were reviewed. Only children with a record of 2 years or more were included as study subjects. All types of cataracts (congenital/developmental, traumatic, and complicated cataracts) and all types of implantations of IOL were included. Thirty-three children were excluded with traumatic cataracts with corneal lacerations or retinal detachment (12 patients) and complicated cataracts associated with other ocular anomalies in the cornea, optic nerve, or retina (21 patients). Therefore, 114 eyes of 57 people were finally selected and analyzed in this study.

The ophthalmologic details and medical histories were investigated for this study. The age at diagnosis and surgery, sex, birth history, family history of congenital cataracts, the type and bilaterality of the cataract, the details of surgical procedures, and postoperative complications were reviewed. Each participant underwent a comprehensive preoperative ophthalmologic assessment, which included the measurement of best-corrected visual acuity (BCVA), intraocular pressure, refractive error (demonstrated as spherical equivalent, SE), keratometry (K), axial length (AL), slit-lamp biomicroscopy, and fundus examination. Visual acuity was expressed based on the logMAR scale (Jin’s vision chart, Seoul, Korea, ISO 8596). To predict and calculate the IOL power, preoperative AL and keratometry were measured either prior to surgery or at the time of surgery. Cooperative children were examined using an auto kerato-refractometer (RK-F10; Canon, Tochigi-ken, Japan) and a manual keratometer (OM-4; Topcon, Tokyo, Japan). AL measurements were performed with the IOL Master (Carl Zeiss AG, Oberkochen, Germany) or by ultrasound biometry (Storz Compuscan; Storz Ophthalmic Inc., St. Louis, MO, USA). Intraoperative evaluation under general anesthesia was performed on infants and toddlers who could not cooperate with the above examinations, using a handheld auto kerato-refractometer (Retinomax K-plus 2; Mandarin Opto-Medic Co., Singapore). IOL power was calculated using 4 different IOL formulas (Saunders Retslaff Kraft (SRK) II, SRK T, Holladay I, and Hoffer Q). The target postoperative refraction was predicted and modified by the subject’s age and the refractive error of the fellow eye, based on the results from previous studies in our institution [28, 29].

All cataract surgeries were performed with a standardized technique. A 3-mm scleral corneal incision was made, then an anterior capsulotomy was done by continuous curvilinear capsulorhexis (CCC) or “can-opener” capsulotomy. Irrigation/aspiration of the lens cortex and nucleus followed. Posterior CCC with anterior vitrectomy was generally performed on patients younger than five years of age at the time of surgery. IOL insertion in the capsular bag or sulcus was done according to the subject’s age or lens capsular status.

Postoperative data on the refractive error, keratometry, and AL of all patients were collected one month and one year after IOL implantation, and then yearly to measure the change in ocular biometry of the pseudophakic eyes. The predictive error using SRK II formula was calculated as absolute error between target and actual postoperative one month refraction. The myopic shift (SE change), axial eye growth (AL change), and corneal curvature (steep K and flat K) of the pseudophakic eyes were compared with the outcome data of the fellow phakic eyes at each yearly follow-up visit based on the one-month postoperative values to analyze the ocular growth following unilateral pediatric cataract surgery. The overall myopic shift and axial eye growth of the pseudophakic eyes and fellow eyes during the total follow-up period were measured and compared for each age group. The subjects were classified into four groups according to their age at IOL implantation: under 3 years (infant/toddler period, 6 eyes), 3 to 5 years (preschool period, 14 eyes), 6 to 8 years (early-school period, 18 eyes), and over 9 years of age (late-school period, 19 eyes) to analyze the age-associated ocular biometric changes.

The data are expressed as means ± standard deviations or numbers (percentages) as appropriate. Demographics were compared using the Mann-Whitney U test and Kruskal-Wallis H test or chi-squared test for continuous variables and categorical variables, respectively. The Wilcoxon signed-rank test was used for comparing data at baseline and the follow-up visits. Simple linear regression analysis using 10 years of postoperative data was performed to determine the myopic shift trends in each age group. All statistical analyses were performed using statistical analysis software (SPSS version 24.0; IBM Corp., Armonk, NY, USA). A P-value of less than 0.05 was considered to be statistically significant.

## Results

A total of 114 eyes (57 pseudophakic eyes and 57 fellow phakic eyes) in 57 children were analyzed in this study. Unilateral cataract was noticed in 45 children, while 12 children with bilateral cataracts had asymmetric lens opacity, and their unoperated eyes were found to have a BCVA of more than 20/40 and did not require cataract extraction. Among the 69 eyes with cataracts, 60 eyes (87.0%) had congenital/developmental cataracts, four eyes (5.8%) had traumatic cataracts without other ocular segments affected, and five eyes (7.2%) had steroid-induced complicated cataracts (Table 1). The most common type of cataract was posterior subcapsular (65.2%), four of them had posterior lenticonus, followed by nuclear/lamellar (28.9%), and anterior subcapsular (5.8%). The detailed surgical procedures and complications after unilateral pediatric cataract surgery are shown in Table 1. A lens aspiration procedure was performed on 57 eyes, 42.1% of which had posterior CCC with anterior vitrectomy. In terms of postoperative complications, 24 eyes (42.1% of all pseudophakic eyes) showed complications of opacification in the posterior capsule (PCO), two eyes (3.5%) developed IOL opacity, two eyes (3.5%) had cortical remnants, and none showed secondary glaucoma following cataract surgery. Posterior capsulotomy was performed in 24 patients with PCO, 20 of whom were able to achieve clear vision after Nd-YAG laser therapy. Surgical pars plana posterior capsulotomy was chosen in the other four patients since they were too young to cooperate with the laser procedure.

**Table 1:** Clinical characteristics of 57 children who underwent unilateral cataract surgery.

Clinical characteristics	No (%)
Male: Female	27: 30
Birth Hx, full-term: preterm	56: 1
Family Hx of congenital cataracts	2 (3.5%)
Bilaterality of cataract	
Bilateral	12 (21.1%)
Unilateral	45 (78.9%)
Cause of cataract (69 eyes)	
Congenital/developmental cataract	60 (87.0%)
Traumatic cataract	4 (5.8%)
Complicated cataract	5 (7.2%)
Type of cataract (69 eyes)	
Anterior subcapsular	4 (5.8%)
Nuclear or lamellar	20 (29.0%)
Posterior subcapsular or posterior lenticonus	45 (65.2%)
Surgical eye, right: left	24: 33
Surgical procedure (57 eyes)	
Posterior CCC with anterior vitrectomy	24 (42.1%)
Primary/secondary IOL implantation	55 (96.5%) / 2 (3.5%)
In the bag / sulcus insertion	50 (87.7%) / 7 (12.3%)
Complications after surgery	
PCO	24 (42.1%)
Nd-Yag laser posterior capsulotomy	20 (35.1%)
Surgical pars plana posterior capsulotomy	4 (7.0%)
IOL opacification	2 (3.5%)
Cortex remnant	2 (3.5%)
High IOP	0 (0.0%)

Hx, history; CCC, continuous curvilinear capsulorhexis; IOL, intraocular lens; PCO, posterior capsular opacity; IOP, intraocular pressure

Table 2 shows the early postoperative biometric change of pseudophakic eyes, at the time of IOL implantation and one month postoperatively. Children with older age had longer AL (P = 0.001), with a mean of 20.43 mm for group I, 22.32 mm for group II, 23.16 mm for group III, and 23.58 mm for group IV. One month after the IOL implantation, significant VA improvement was seen in all age groups. In a comparison of postoperative SE with the preoperative anticipated target using SRK II principles, no statistical differences were found in any age group (P = 0.882).

**Table 2:** Ocular biometric findings of pseudophakic eyes at the time of IOL implantation and one-month postoperatively.

Group	All	I (Infant/toddler) age <3	II (Preschool) 3≤ age <6	III (Early-school) 6≤ age <9	IV (Late-school) age ≥9	P-value
Number of eyes	57	6	14	18	19	
Age at IOL implantation (year)	7.55 ± 3.57	1.77 ± 0.78	4.25 ± 0.72	7.93 ± 0.75	11.46 ± 1.62	<0.001^a^
AL (mm)	22.78 ± 1.47	20.43 ± 1.69	22.32 ± 1.38	23.16 ± 1.08	23.58 ± 0.87	0.001^a^
Mean K (D)						
Preoperative	43.29 ± 1.58	43.79 ± 2.63	42.95 ± 1.85	43.37 ± 1.20	43.36 ± 1.65	0.734
Postoperative 1-month	43.76 ± 1.60	45.50	43.75 ± 1.40	43.58 ± 1.15	43.79 ± 2.36	0.601
SE (D)						
Preoperative	-0.46 ± 4.16	-4.31 ± 7.51	+0.45 ± 3.36	+0.47 ± 4.33	-1.16 ± 4.18	0.278
Postoperative 1-month	+0.50 ± 1.97	+1.92 ± 4.71	+1.25 ± 1.52	+0.49 ± 0.99	-0.50 ± 1.08	0.011^a^
Anticipated target (SRK II)	+0.67 ± 1.45	+3.00 ± 2.28	+1.24 ± 1.18	+0.74 ± 0.64	-0.52 ± 0.90	<0.001^a^
P-value^b^	0.882	0.715	0.861	0.394	0.691	
BCVA (logMAR)						
Preoperative	0.87 ± 0.63	N/A	0.94 ± 0.67	0.73 ± 0.29	0.95 ± 0.82	0.679
Postoperative 1-month	0.37 ± 0.30	0.60	0.57 ± 0.31	0.35 ± 0.21	0.24 ± 0.30	0.003^a^
P-value	<0.001^b^	N/A	0.036^b^	<0.001^b^	<0.001^b^	

AL, axial length; K, keratometry; D, diopter; SE, spherical equivalent; BCVA, best-corrected visual acuity; N/A, not applicable

The data are expressed as means ± standard deviations.

^a^Comparison was performed using the Kruskal Wallis H test. Significant values at P < 0.05.

^b^Postoperative 1-month SE and anticipated target were compared, and preoperative BCVA was compared with postoperative BCVA. Comparison was performed using the Wilcoxon signed-rank test. Significant values at P < 0.05.

**Fig 1:**
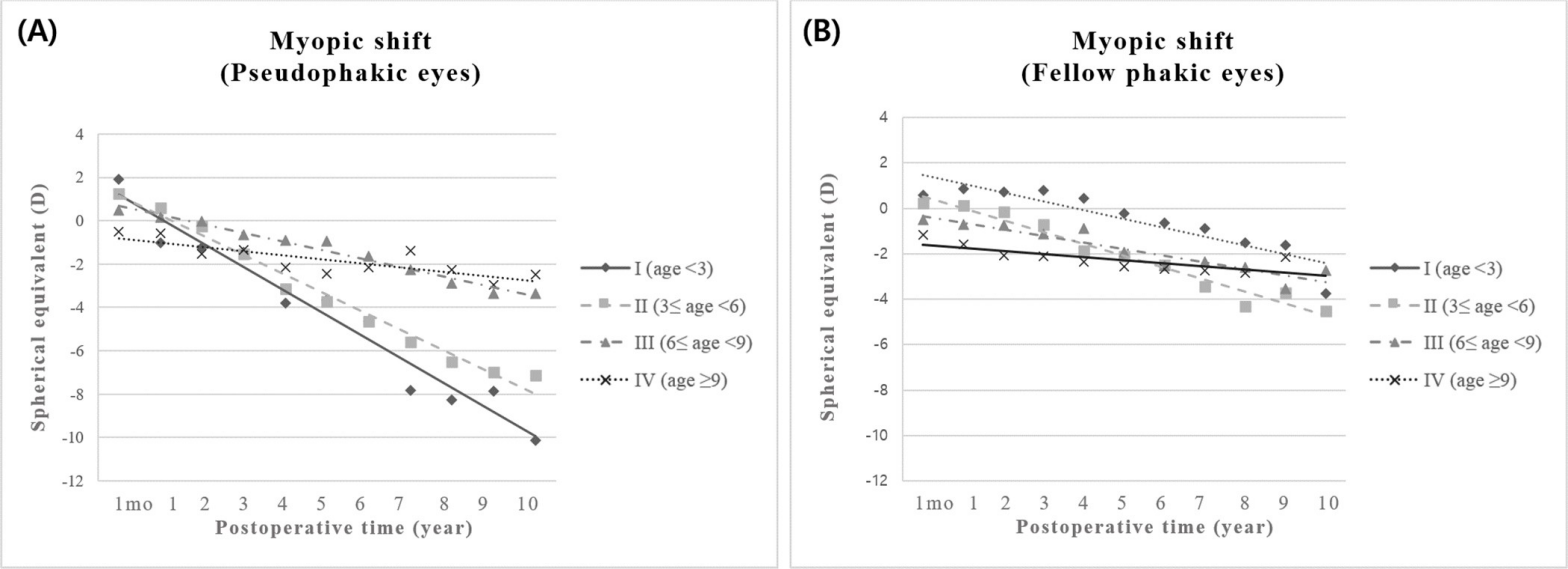
Ten years of spherical equivalent values on the pseudophakic and fellow phakic eyes in the four groups divided by age. (A) Group I had the steepest yearly change in spherical equivalent in the pseudophakic eyes among the four age groups at -1.11 D (R^2^ = 0.954), followed by group II at -0.91 D (R^2^ = 0.979), group III at -0.42 D (R^2^ = 0.975), and group IV at -0.20 D (R^2^ = 0.674) (P < 0.001). (B) No significant difference was shown in yearly SE changes in the phakic eyes among the age groups (-0.39 D in group I; -0.52 D in group II; -0.29 D in group III; and -0.14 D in group IV, P = 0.502).

Long-term yearly biometric data of the subjects were analyzed (Table 3). The mean follow-up period of 57 children was 7.81 (2.2–21.6) years and the final follow-up age was 15.4 ± 6.19 years. During the total follow-up period, the overall myopic shift (D) and the yearly rate of myopic shift (D/year) were significantly higher with -3.25 ± 3.21 D and -0.45 ± 0.44 D/year in the pseudophakic eyes than with -1.78 ± 2.10 D and -0.22 ± 0.29 D/year in the fellow phakic eyes (P = 0.01 and P = 0.004, respectively). In the comparison of each age group, group I and group II showed a more rapid myopic shift in the pseudophakic eyes than in the fellow phakic eyes, as -1.14 ± 0.66 D/year versus (vs.) -0.02 ± 0.45 D/year in group I (P = 0.009) and -0.63 ± 0.37 D/year vs. -0.31 ± 0.29 D/year in group II (P = 0.014). The amount and rate of myopic shift in the pseudophakic eyes decreased in the older age groups (P = 0.001), and no significant between-eye difference in the myopic shift was found in children who underwent unilateral cataract surgery at the age of 6 or older. Ten years of SE data on the pseudophakic and fellow phakic eyes in the four groups divided by age are shown in Fig 1. Using linear regression analysis, group I was revealed to have the steepest yearly change in SE (R^2^ = 0.954) among the 4 age subgroups of pseudophakic eyes with -1.11 D/year, followed by group II at -0.91 D/year (R^2^ = 0.979), group III at -0.42 D/year (R^2^ = 0.975), group IV at -0.20 D/year (R^2^ = 0.674) (P < 0.001, Fig 1A). However, the phakic eyes did not show any significant difference in yearly SE changes in any age subgroup (P = 0.502, Fig 1B).

**Table 3:** Comparison of myopic shift and axial eye growth of pseudophakic eyes and fellow phakic eyes during the total follow-up period after unilateral pediatric cataract surgery.

Spherical equivalent change							
Group (age at IOL implantation)	Follow-up period (year)	Overall myopic shift (D)			Rate of myopic shift (D/year)		
		Pseudophakic eye	Phakic eye	P-value	Pseudophakic eye	Phakic eye	P-value
All	7.81 ± 4.39	-3.25 ± 3.21	-1.78 ± 2.10	0.01^a^	-0.45 ± 0.44	-0.22 ± 0.29	0.004^a^
I (age <3)	6.65 ± 3.36	-6.46 ± 4.12	-0.52 ± 2.09	0.009^a^	-1.14 ± 0.66	-0.02 ± 0.45	0.009^a^
II (3≤ age <6)	6.94 ± 3.05	-5.07 ± 3.91	-2.46 ± 2.82	0.056	-0.63 ± 0.37	-0.31 ± 0.29	0.014^a^
III (6≤ age <9)	8.13 ± 5.28	-2.43 ± 2.00	-1.93 ± 1.98	0.496	-0.30 ± 0.21	-0.23 ± 0.30	0.339
IV (age ≥9)	8.52 ± 4.70	-1.62 ± 1.63	-1.55 ± 1.43	0.908	-0.23 ± 0.27	-0.21 ± 0.20	0.977
P-value		0.001^b^	0.429		<0.001^b^	0.502	
Axial length change							
Group (age at IOL implantation)	Follow-up period (year)	Overall axial eye growth (mm)			Rate of axial eye growth (mm/year)		
		Pseudophakic eye	Phakic eye	P-value	Pseudophakic eye	Phakic eye	P-value
All	6.33 ± 2.98	1.49 ± 1.14	1.42 ± 0.91	0.94	0.23 ± 0.16	0.26 ± 0.15	0.504
I (age <3)	7.35 ± 3.02	2.83 ± 1.16	2.15 ± 1.69	0.486	0.32 ± 0.07	0.25 ± 0.17	0.886
II (3≤ age <6)	6.26 ± 2.90	2.11 ± 1.34	1.66 ±0.93	0.512	0.33 ± 0.15	0.29 ± 0.10	0.468
III (6≤ age <9)	6.02 ± 3.19	0.98 ± 0.51	1.12 ± 0.53	0.606	0.17 ± 0.12	0.25 ± 0.19	0.243
IV (age ≥9)	6.32 ± 2.92	0.88 ± 0.65	0.99 ± 0.29	0.743	0.18 ± 0.18	0.23 ± 0.18	0.583
P-value		0.006^b^	0.494		0.015^b^	0.787	

IOL, intraocular lens; D, diopter

The data are expressed as means ± standard deviations.

^a^Comparison was performed using the Mann-Whitney U test. Significant values at P < 0.05.

^b^Comparison was performed using the Kruskal Wallis H test. Significant values at P < 0.05.

Long-term postoperative changes in axial length were also evaluated (Table 3). Younger age groups had a larger amount and higher yearly rate of axial growth in the pseudophakic eyes. The overall axial growth was more prominent in the younger age groups in the pseudophakic eyes (P = 0.006), whereas all age groups showed similar axial growth patterns in the phakic eyes (P = 0.494). However, neither the overall amount nor the yearly rate of axial eye growth showed any significant difference between the pseudophakic and phakic eyes (P = 0.94 and P = 0.504, respectively), even in groups I and II that showed significant myopic shifts in the pseudophakic eyes.

The keratometry values of the pseudophakic and phakic eyes at postoperative one month and at final follow-up are shown in Table 4. No statistical differences between the eyes or among the age groups were found. Corneal astigmatism (steep K–flat K difference) increased over time at 0.56 ± 0.83 D in the pseudophakic eyes vs. 0.36 ± 0.69 D in the phakic eyes, but no significant between-eye difference was found (P = 0.559).

**Table 4:** Keratometry values of pseudophakic and fellow phakic eyes at postoperative one-month and at final follow-up after unilateral pediatric cataract surgery.

Group (age at IOL implantation)	Steep K (D) at postop. 1-month			Flat K (D) at postop. 1-month		
	Pseudophakic eye	Phakic eye	P-value^a^	Pseudophakic eye	Phakic eye	P-value^a^
All	44.78 ± 1.77	43.87 ± 1.39	0.058	42.73 ± 1.63	42.57 ± 1.60	0.671
I (age <3)	47.50	45.25	N/A	43.50	43.00	N/A
II (3≤ age <6)	44.44 ± 1.66	43.90 ± 1.83	0.556	43.06 ± 1.14	42.80 ± 1.81	0.73
III (6≤ age <9)	44.59 ± 1.23	44.08 ± 1.16	0.173	42.57 ± 1.37	42.63 ± 1.45	0.809
IV (age ≥9)	44.89 ± 2.48	43.15 ± 1.43	0.202	42.68 ± 2.39	42.15 ± 2.09	0.755
P-value^b^	0.517	0.4		0.769	0.988	
Group (age at IOL implantation)	Steep K (D) at final follow-up			Flat K (D) at final follow-up		
	Pseudophakic eye	Phakic eye	P-value^a^	Pseudophakic eye	Phakic eye	P-value^a^
All	44.37 ± 2.09	43.68 ± 1.94	0.096	41.93 ± 1.94	42.18 ± 1.86	0.772
I (age <3)	44.93 ± 1.84	43.75 ± 1.41	0.533	42.74 ± 1.56	41.88 ± 1.59	0.533
II (3≤ age <6)	44.74 ± 2.11	44.48 ± 3.06	0.927	42.60 ± 2.25	42.42 ± 2.53	1.0
III (6≤ age <9)	44.33 ± 1.49	43.66 ± 1.63	0.21	41.89 ± 1.50	42.38 ± 1.62	0.547
IV (age ≥9)	44.02 ± 2.74	43.33 ± 1.95	0.471	41.37 ± 2.26	41.97 ± 2.09	0.695
P-value^b^	0.733	0.659		0.559	0.707	

IOL, intraocular lens; K, keratometry; D, diopter; postop., postoperative day; K-diff, difference in steep-flat K; N/A, not applicable

The data are expressed as means ± standard deviations.

^a^Comparison was performed using the Mann-Whitney U test. Significant values at P < 0.05.

^b^Comparison was performed using the Kruskal Wallis H test. Significant values at P < 0.05.

## Discussion

This study investigated the long-term ocular biometric changes between pseudophakic and the fellow phakic eyes after unilateral pediatric cataract surgery with an average follow-up of 7.81 years. Following unilateral cataract surgery, the pseudophakic eyes showed a higher overall amount and rate of myopic shift than the fellow phakic eyes, with the absence of any significant between-eye differences in axial length and keratometric values. A significant postoperative myopic shift was found in children who underwent unilateral cataract surgery under 6 years of age. These findings have an important clinical implication that postoperative myopic shift and the resultant anisometropia should be considered when setting the target refraction and selecting the optimal IOL power at the time of unilateral cataract surgery in children.

Refractive changes following unilateral pediatric cataract surgery have been investigated in several studies (Table 5). Some studies with different ethnicities have demonstrated a greater myopic shift in pseudophakic eyes [4, 6, 11, 16]. They found a more rapid myopic shift of -0.65 to -2.12 D/year in pseudophakic eyes compared to -0.12 to -0.40 D/years in the fellow eyes. However, a Taiwanese study [17] with a mean age of 5.6 years at surgery did not find any significant difference in refractive changes between the pseudophakic eyes and the fellow phakic eyes. In a study by Sminia et al. [11], there was no difference in an older age group at a mean of 4.1 years. The results of the present study revealed a significantly higher rate of refractive change in the pseudophakic eyes at an average of -0.45 D/year, compared to -0.22 D/year in the fellow phakic eyes. The subgroup analysis according to age at surgery showed that children who underwent cataract surgery earlier had a larger (groups I and II) and more rapid (group I) postoperative myopic shift. The overall amount and myopic shift rate in the pseudophakic eyes decreased in the older age groups (P = 0.001), and no significant between-eye difference in myopic shift was found in children who underwent unilateral cataract surgery at the age of 6 or older. In particular, a postoperative myopic shift was noticeable in children with an IOL implanted before 3 years of age. They showed a mean myopic shift of -6.46 ± 4.12 D in the pseudophakic eyes and -0.52 ± 2.09 D in the fellow phakic eyes during a mean follow-up period of 6.65 years after cataract surgery. These findings imply that children with unilateral pseudophakia who underwent cataract surgery earlier are at risk of developing significant anisometropia due to a large myopic shift occurring in the pseudophakic eyes. A recent prospective study, which included infants who underwent unilateral IOL implantation at a median age of 2.2 months, pointed out that the majority of them had significant anisometropia at an age of 5 years [16].

**Table 5:** Previous studies on refractive changes after unilateral pediatric cataract surgery.

References	Ethnic population	No. of eyes	Age at IOL implantation (years), mean (range)	Mean rate of refractive change (D/year)		Follow-up years, mean (range)
				Pseudophakic eye	Fellow eye	
Enyedi et al. ^b^ [4]	United States	83	6.3 (0.75–17)	-0.65	-0.12	2.2 (0.5–6.6)
				(-1.2 in age≤2)		
Yam et al. ^b^ [6]	Hong Kong Chinese	32	7.3 (1.5–17)	-0.84	-0.24	4.4 (2–9)
				(-0.91 in age≤2)		
Sminia et al. ^a^^,^ ^b^ [11]	Netherlands	20	4.1 (2.2–8)	-0.21	-0.23	4.8 (1.8–11.1)
		25	0.4 (0.06–1.4)	-1.40^b^	-0.40	4.3 (1.0–11.9)
Weakley et al.* [16]	United States	108	0.2 (0.08–0.6)	-2.12	-	4.9 (0.9–5.4)
Yang et al. [17]	Taiwanese	62	5.6 (1–9.8)	-0.46	-0.23	3.6 (1–9.75)
Present study^b^	Korean	114	7.6 (0.5–15.6)	-0.45	-0.22	7.8 (2.2–21.6)
				(-1.14 in age<3)		

IOL, intraocular lens; D, diopter

*The Infant Aphakia Treatment Study

^a^ The patients were divided into two groups based on an age of 18 months.

^b^ Studies with different ethnicities that have demonstrated a significantly higher rate of refractive change (D/year) in pseudophakic eyes than in fellow phakic eyes.

We could not find any significant between-eye differences in the changes of axial length and keratometric values following unilateral cataract surgery, in contrast to the large myopic shift observed in the unilateral pseudophakic eyes. Differences in the amount and yearly rate of axial eye growth showed no statistical significance between the pseudophakic and phakic eyes (P = 0.94 and P = 0.504, respectively) in any age subgroup. Similar findings were found in previous studies [11, 12, 30], these findings provide supportive evidence that the myopic shift in the operated eye is an optical phenomenon. In developing phakic eyes, progressive flattening of the crystalline lens decreases the refractive consequences of axial elongation [19, 20]. After lens removal, a myopic shift in pseudophakic eyes could occur if the fixed power of the IOL fails to offset the axial growth. As the eye grows, the IOL moves farther from the retina. Similar to the effect of vertex distance with high-power lenses, the anterior movement of the IOL as the child grows induces a myopic shift by itself [12, 30]. Our study found that the younger age groups showed more rapid axial eye growth compared to the older age groups, consistent with the previous studies [13, 15, 19, 31, 32]. Although there were no between-eye differences in axial elongation following surgery, the large axial growth observed in younger children might lead to significant refractive changes in the pseudophakic eyes with fixed IOL power. Moreover, the incidence and severity of myopia have been increasing in several Asian countries, including Korea. A recent population-based study reported that the prevalence rates of myopia (≤ −0.5 D) and high myopia (≤ −6.0 D) among 13–18-year-old Korean adolescents were 80.1% and 8.9%, respectively [33]. These ethnic variations could make a greater impact on the pattern of long-term myopic shifts in Korean children and require further research.

IOL selection is still challenging in growing children who do not have a static course after cataract extraction. It is more difficult to choose targeted refraction in children who require unilateral cataract surgery to minimize anisometropia and the following amblyopia and prevent the future development of high myopia in the long perspective. An analysis of the long-term refractive change of unilateral pseudophakia enables us to anticipate a myopic shift developing after surgery. Kraus et al. [34] reported 10 cases that required an IOL exchange for high myopia in children with unilateral pseudophakia. A recent randomized clinical trial of the Infant Aphakia Treatment Study group (IATS) [16] revealed significant anisometropia with a median value of -3.5 D in 5-year-old children after unilateral IOL implantation in infants. The authors found that the immediate postoperative refractive targets of +8 D or +6 D were not sufficient to compensate for the actual myopic shifts observed in that study [16]. The major findings in the present study were: (1) more rapid myopic shift in pseudophakic eyes in children aged 6 years and younger at the time of cataract surgery, (2) approximately -6.0 D greater myopic shift in the pseudophakic eyes than in the fellow phakic eyes at an average follow-up of 6.65 years in children younger than 3 years of age at surgery, and (3) no significant between-eye difference in the overall amount and rate of myopic shift in children who underwent unilateral cataract surgery at age over 6 years old. These findings should be considered differently depending upon the age at surgery in optimizing IOL selection in children requiring unilateral cataract surgery. For children older than 6 years of age, an IOL power could be selected with the target refraction matched to the refractive error of the fellow eye. For younger children under 6 years of age, since they are susceptible to developing amblyopia, efforts should be given to facilitate visual development in the treated pseudophakic eye. According to the 2018 American Academy of Ophthalmology (AAO) guidelines [35], the threshold for the correction of aniso-hypermetropia was +2.5 D for children younger than one year, +2.0 D for children 1–2 years old, and +1.5 D for children older than 2 years. Also, in a long-term perspective, the power of an IOL should be selected that will not develop significant anisometropia and high myopia following the period of ocular growth [34]. Based on the large myopic shift observed in young children, it could be reasonable to determine an optimal IOL power of < +2.0 D more hyperopic than the refractive error of the fellow phakic eye. These guidelines could be practically useful in treating children who require unilateral cataract surgery due to acquired cataracts related to trauma or drugs as well as congenital causes.

There were some limitations to our study. First, since the causes and types of cataracts among patients enrolled were diverse, it may have influenced the results to some extent. Second, the predictive error was calculated using the SRK-II formula only. Third, our series was performed retrospectively and had relatively small subject numbers in some subgroups. The younger age groups did not have sufficient numbers of children to perform a parametric statistical analysis. Limited numbers of patients have undergone unilateral cataract extraction and primary IOL implantation before 3 years old due to the complexity of the surgical techniques and the risk of various intraoperative and postoperative complications [36]. Further prospective studies with a larger sample size and long-term follow-up are required to confirm the results observed in the present study, especially focusing on very young children.

## Conclusion

A significant postoperative myopic shift was noticed in pseudophakic eyes following unilateral cataract surgery compared to the fellow phakic eyes, especially in young children under 6 years of age. Axial length and keratometric changes did not accompany a myopic shift. Pediatric cataract surgeons should be aware of the long-term ocular biometric changes developed in pseudophakic eyes during ocular growth following cataract surgery.

## Supporting information

S1 Data Study data. All patient results and demographics information.
(XLSX)
